# Use of Anthrax Vaccine in the United States: Recommendations of the Advisory Committee on Immunization Practices, 2019

**DOI:** 10.15585/mmwr.rr6804a1

**Published:** 2019-12-13

**Authors:** William A. Bower, Jarad Schiffer, Robert L. Atmar, Wendy A. Keitel, Arthur M. Friedlander, Lindy Liu, Yon Yu, David S. Stephens, Conrad P. Quinn, Katherine Hendricks

**Affiliations:** ^1^Division of High-Consequence Pathogens and Pathology, National Center for Emerging and Zoonotic Infectious Diseases, CDC; ^2^Division of Bacterial Diseases, National Center for Immunization and Respiratory Diseases, CDC; ^3^Department of Medicine, Baylor College of Medicine, Houston, Texas; ^4^Department of Molecular Virology & Microbiology and Department of Medicine, Baylor College of Medicine, Houston, Texas; ^5^U.S. Army Medical Research Institute of Infectious Diseases, Frederick, Maryland; ^6^Division of Preparedness and Emerging Infections, National Center for Emerging and Zoonotic Infectious Diseases, CDC; ^7^Department of Medicine, Emory University, Atlanta, Georgia; ^8^Office of Laboratory Science, CDC

## Abstract

This report updates the 2009 recommendations from the CDC Advisory Committee on Immunization Practices (ACIP) regarding use of anthrax vaccine in the United States (*Wright JG, Quinn CP, Shadomy S, Messonnier N. Use of anthrax vaccine in the United States: recommendations of the Advisory Committee on Immunization Practices [ACIP)], 2009. MMWR Recomm Rep 2010;59[No. RR-6]*). The report 1) summarizes data on estimated efficacy in humans using a correlates of protection model and safety data published since the last ACIP review, 2) provides updated guidance for use of anthrax vaccine adsorbed (AVA) for preexposure prophylaxis (PrEP) and in conjunction with antimicrobials for postexposure prophylaxis (PEP), 3) provides updated guidance regarding PrEP vaccination of emergency and other responders, 4) summarizes the available data on an investigational anthrax vaccine (AV7909), and 5) discusses the use of anthrax antitoxins for PEP.

Changes from previous guidance in this report include the following: 1) a booster dose of AVA for PrEP can be given every 3 years instead of annually to persons not at high risk for exposure to *Bacillus anthracis* who have previously received the initial AVA 3-dose priming and 2-dose booster series and want to maintain protection; 2) during a large-scale emergency response, AVA for PEP can be administered using an intramuscular route if the subcutaneous route of administration poses significant materiel, personnel, or clinical challenges that might delay or preclude vaccination; 3) recommendations on dose-sparing AVA PEP regimens if the anthrax vaccine supply is insufficient to vaccinate all potentially exposed persons; and 4) clarification on the duration of antimicrobial therapy when used in conjunction with vaccine for PEP.

These updated recommendations can be used by health care providers and guide emergency preparedness officials and planners who are developing plans to provide anthrax vaccine, including preparations for a wide-area aerosol release of *B. anthracis* spores. The recommendations also provide guidance on dose-sparing options, if needed, to extend the supply of vaccine to increase the number of persons receiving PEP in a mass casualty event.

## Summary

## Introduction

Anthrax is an acute febrile illness caused by infection with *Bacillus anthracis*. The mortality rate, even with treatment, ranges from <2% for cutaneous anthrax (*1*) to 45% for inhalation anthrax (*2*) and 92% for anthrax meningitis (*3*). *B. anthracis* is a zoonotic pathogen that primarily infects sheep, goats, cattle, and other herbivores. Humans become infected after exposure to infected animals or contaminated animal products or, rarely, as a complication from injection drug use (*4*). *B. anthracis* is also a tier 1 select agent and is considered one of the bioterrorism agents that is most likely to be used because it is relatively easy to acquire from the natural environment, mass produce, and disseminate as spores via aerosolization (*5*).

Anthrax vaccine adsorbed (AVA) (BioThrax) is licensed for preexposure prophylaxis (PrEP) for adults aged 18–65 years at high risk for exposure to *B. anthracis* (*6*). The dosage approved by the U.S. Food and Drug Administration (FDA) is 0.5 mL administered intramuscularly (IM) at 0, 1, and 6 months with boosters at 6 and 12 months after completion of the primary series and at 12-month intervals thereafter. AVA also is licensed for postexposure prophylaxis (PEP) in combination with antimicrobials for adults aged 18–65 years with suspected or known exposure to aerosolized *B. anthracis* spores. The dosage approved by FDA is 0.5 mL administered subcutaneously (SC) at 0, 2, and 4 weeks. For persons not included in the FDA-approved indication for PEP, AVA will be available for PEP use for children, pregnant women, nursing mothers and older adults (i.e., ≥66 years) under appropriate emergency use regulatory provisions. Although data are lacking on the immune impact of mixing the IM and SC routes of administration, as might occur when switching from PrEP to PEP, switching between routes would be unlikely to adversely impact immunity because both routes provide adequate immunity.

AV7909 (AVA plus CpG 7909 adjuvant) is a second-generation anthrax vaccine produced by Emergent BioSolutions that is in a phase 3 trial (https://clinicaltrials.gov). The CpG 7909 adjuvant binds to the Toll-like receptor 9 to enhance the immune response to coadministered antigens (primarily *B. anthracis* protective antigen) (*7*,*8*). The PEP schedule, under clinical evaluation for AV7909, is 0.5 mL AVA with 0.25 mg CpG 7909 adjuvant administered IM at 0 and 2 weeks postexposure, combined with antimicrobials.

Since the publication in 2010 of the Advisory Committee on Immunization Practices (ACIP) recommendations for use of anthrax vaccine in the United States (*9*), published studies have 1) addressed the efficacy, immunogenicity, and reactogenicity of the recommended and alternate dose-sparing schedules of AVA; 2) estimated AVA efficacy in humans from data on animal efficacy and human antibody levels by using a correlates of protection model; and 3) evaluated whether developing chronic illnesses or having adverse pregnancy outcomes are associated with previous AVA receipt. In addition, AV7909 phase 1 and 2 clinical trials have demonstrated the potential of AV7909 for use as the vaccine component of PEP (PEP-Vx) (*10*–*12*).

Given these newly available data, CDC asked ACIP to revise the recommendations for use of anthrax vaccines in the United States. These revised recommendations address the IM versus SC administration of AVA for PEP and the use of reduced-schedule and half-dose AVA during public health emergencies, shortening the duration of antimicrobials given in conjunction with PEP-Vx, and extending the AVA PrEP booster dose interval after the initial priming and booster series. This report provides recommendations and guidance regarding the use of AVA for PrEP and PEP and updates the ACIP anthrax vaccination recommendations published in 2002 and 2010. This report also describes available data for AV7909 because of its potential for prelicensure emergency use during a large-scale anthrax public health emergency if the AVA supply is inadequate. This report can be used by health care providers to update the current practice for providing anthrax vaccine for PrEP and PEP and can be used by emergency preparedness partners to develop emergency vaccine response plans in preparation for a wide-area aerosolized release of *B. anthracis* spores.

## Methods

During March 2017–January 2019, the ACIP Anthrax Vaccines Work Group (AVWG), which comprises professionals from academic medicine (internal medicine, pediatrics, obstetrics, and infectious disease specialists), federal and state public health entities, and medical societies, participated in monthly telephone conferences facilitated by CDC. During these meetings, AVWG reviewed relevant scientific evidence and evaluated the quality of the evidence assessing the 1) immunogenicity and safety of an extended booster dose interval for PrEP in persons not at high risk for exposure to *B. anthracis* but who might have a future high risk for exposure; 2) benefits and harms of the IM versus SC route of administration for PEP-Vx; 3) benefits and harms of AVA dose-sparing schedules (i.e., 2 full doses or 3 half doses) for PEP-Vx if vaccination demands were to exceed vaccine supply after a wide-area aerosolized release of *B. anthracis* spores; 4) immunogenicity and safety of AV7909, based on available data; and 5) use of anthrax antitoxin for PEP in conjunction with anthrax vaccine.

A scientific literature search was conducted through a systematic review for studies involving human subjects or for animal studies that met criteria for the Animal Rule (*13*,*14*) that reported primary data on important health outcomes related to AVA or AV7909 published after 2008. The previous ACIP review summarized the data through 2008 (*9*). Databases searched in February 2017 included Medline (OVID), Embase (OVID), CAB Abstracts (OVID), Global Health (OVID), CINAHL (Ebsco), Econlit (Ebsco), Cochrane Library, Clinical Trials.gov, FedRip (Ebsco), the U.S. Department of Defense (DoD) Technical Information Center, NTIS:NTRL, Scopus, WHOLIS, and WorldCat. Search terms included anthrax vaccine, AVA, Biothrax, Nuthrax, AV7909, CpG DNA, CpG 7909, CpG motifs, CpG oligodeoxynucleotide, Anthim, Anthrasil, obiltoxaximab, and raxibacumab. In addition, the work group reviewed unpublished data from the CDC Anthrax Vaccine Research Program, unpublished data from the vaccine manufacturer, and results of studies from the Vaccine Analytic Unit, which is a CDC-led collaboration with DoD and FDA that assessed potential associations of AVA with development of chronic conditions (*15*). The review of vaccine safety also included adverse events reported to the Vaccine Adverse Event Reporting System (VAERS) after AVA administration for January 1, 2009, through June 30, 2017 (*16*). To qualify as a candidate for inclusion in the review, a study had to present immunogenicity or safety data on AVA, AV7909, or infectious disease vaccines that used CpG 7909 adjuvant. Studies were excluded if they lacked mention of either AVA or AV7909 for the prevention of anthrax, lacked primary data, or were outside the time frame of interest.

Data were abstracted and summarized for immunogenicity outcomes of interest, including seroconversion, geometric mean concentration (GMC) of anti-protective antigen immunoglobulin G (anti-PA IgG, determined by enzyme-linked immunosorbent assay), geometric mean titer (GMT) of toxin neutralization activity (TNA), effective dose 50 (ED_50_) levels, and GMT of TNA neutralization factor 50 (NF_50_) levels. Data also were abstracted and summarized for safety outcomes of interest, including injection site adverse events, systemic adverse events, and serious adverse events. Quality of evidence was evaluated and presented in tabular format using the Grading of Recommendations Assessment, Development and Evaluation (GRADE) approach (*17*).

Evidence that had been summarized for and reviewed by AVWG was publicly presented at the ACIP meetings in June 2017, October 2017, February 2018, June 2018, October 2018, and February 2019 (*18*). After a public comment period, ACIP voting members at the June 2018 and February 2019 meetings unanimously approved the proposed recommendations.

## Risk for Exposure to Anthrax

The risk for exposure to aerosolized *B. anthracis* spores in the United States is very low (*19*). Anthrax is only endemic in a few sparsely populated areas in the western United States (*20*). Certain occupations and other activities place persons at higher risk for exposure (*21*). These include laboratory work that involves bioproduction of large quantities, volumes, or high concentrations of *B. anthracis* spores and activities with a high potential for exposure to aerosolized *B. anthracis* spores, such as military deployment to areas designated by DoD as posing a high risk for anthrax exposure and emergency response activities after release of *B. anthracis* spores (*9*).

The possibility exists of an intentional wide-area aerosolized release of *B. anthracis* spores over a densely populated area in the United States. In 2001, letters containing *B. anthracis* spores sent through the U.S. Postal Service led to 22 cases of anthrax, five of which were fatal (*22*). In addition, certain countries and terrorist groups have explored the use of anthrax as a bioweapon (*23*–*25*). An aerosolized release of *B. anthracis* spores over densely populated areas could become a mass-casualty incident (*26*). However, previously developed, publicly available clinical recommendations only addressed clinical management using conventional standards of care (*27*). To prepare for the possibility of an anthrax mass-casualty incident, when the number of patients is likely to exceed the ability of the health care infrastructure to provide conventional standards of care and supplies might not meet demand, the U.S. government has stockpiled equipment and therapeutics (i.e., medical countermeasures) for anthrax prevention and treatment and provided recommendations for their use (*28*). The U.S. government’s Strategic National Stockpile stores anthrax vaccine to be used with antimicrobials for PEP of persons with known or potential exposure to *B. anthracis* spores, as well as therapeutics and supplies for anthrax treatment. Animal models have shown that although 5–30 days of antimicrobials might be insufficient to prevent anthrax after single exposures or reexposures to *B. anthracis* spores, the addition of vaccine substantially enhances efficacy (*29*–*31*). In the event of a large-scale release of *B. anthracis* spores, the Strategic National Stockpile will distribute medical countermeasures to affected states, and state and local public health agencies will then dispense antimicrobials to and vaccinate numerous at-risk persons. Antimicrobials are given long enough (up to 60 days) to prevent infection until the vaccine can elicit a protective immune response (*29*).

## Summary of Key Findings

### Anthrax Vaccine Adsorbed

Because human efficacy studies of inhalation anthrax are unethical, the effectiveness of AVA for PEP cannot be directly assessed in humans. For this situation, FDA allows the use of the Animal Rule, a set of regulations that allow approval of products critical for the protection of public health and national security based on efficacy data only in animals combined with immunogenicity and safety data in animals and humans (*32*). Under the Animal Rule, AVA vaccine-induced antibody levels were extrapolated from vaccine efficacy studies conducted in animals to predict vaccine effectiveness in humans (*33*). Statistical modeling was used to establish the relation between survival of AVA-vaccinated animals challenged with *B. anthracis* spores and their antibody levels at the time of infectious challenge. This relation was applied to postvaccination antibody levels in humans to estimate the probability of human survival at selected time points (*34*).

#### Route of Administration and Immunogenicity of AVA for PEP

The SC route of administration of AVA is preferred in adults because SC administration results in higher antibody concentrations by week 4 than the IM route: males SC, 40.8 *μ*g/mL (95% confidence interval [CI]: 34.0–49.1); males IM, 26.3 *μ*g/mL (95% CI: 21.9–31.2); females SC, 60.2 *μ*g/mL, (95% CI: 50.1–72.3), females IM, 36.0 *μ*g/ml (95% CI: 30.0–43.1 (*35*). Using these antibody concentrations, survival estimates based on the correlates of protection model are 3.8% higher for the SC route (92.4%) than the IM route (88.6%) of administration at week 4 (*36*). However, by week 9, the antibody concentrations and predicted survival from the IM route (95.6%) and SC route (96.1%) are no longer significantly different (*35*).

In a wide-area aerosolized release of *B. anthracis* spores over a densely populated area, potentially hundreds of thousands of exposed persons might require PEP-Vx to prevent inhalation anthrax. In such a situation, rapid and efficient administration of vaccine to large numbers of persons would be a key component of the public health emergency response. Health care providers typically have more experience administering vaccines by the IM route than the SC route. In addition, during a conference call with state and local jurisdictions, many public health officials indicated that they plan to use just-in-time training for responding vaccinators during a wide-area aerosolized release of *B. anthracis* spores. Officials also stated during this call that training vaccinators to use the IM route was the easiest (State and local public health preparedness officials, personal communication, 2018). In addition, a study comparing the IM and SC route for AVA administration found significantly less reactogenicity (less injection site warmth, itching, erythema, induration, swelling, and nodule formation) with the IM route than with the SC route at 0, 2, and 4 weeks. In this same study, only two adverse events were more common among IM AVA recipients than SC AVA recipients: limitation of arm motion and generalized myalgia (*35*). Because the preponderance of injection site adverse events was associated with the SC route, concern has been raised that using this route might decrease the likelihood of patients completing the second and third doses of AVA.

#### Dose-Sparing Strategies for PEP-Vx

A wide-area aerosolized release of *B. anthracis* spores over a densely populated area could potentially require PEP-Vx of more persons than could be vaccinated with the available supply of AVA in the Strategic National Stockpile if AVA were to be administered according to the licensed regimen (0.5 mL at 0, 2, and 4 weeks) for the PEP-Vx indication. If demand were to exceed the supply, alternative AVA dose-sparing regimens might be needed to provide PEP-Vx to all persons with suspected or known exposure to aerosolized *B. anthracis* spores. To address this problem, studies were reviewed that estimated survival with AVA administered according to the licensed PEP-Vx schedule (3 full [0.5-ml] doses at 0, 2, and 4 weeks) versus alternate dose-sparing schedules (i.e., 2 full doses at 0 and 2 weeks, 2 full doses at 0 and 4 weeks, and 3 half [0.25-ml] doses at 0, 2, and 4 weeks) (Figure). The three groups who received an AVA dose at week 2 had higher antibody concentrations at week 4 than the one group who did not. The dose-sparing schedule of 2 full doses administered 4 weeks apart produced the highest antibody concentrations from week 6 onward after the first dose. The 3 full-dose regimen produced higher antibody concentrations than when the vaccine was administered as 3 half doses at all measured time points after week 1. The peak response was measured 2 weeks after the last dose for the licensed and dose-sparing PEP-Vx schedules and was estimated to be highly protective by the correlates of protection model (*37*). The predicted survival was estimated to be 97.4% (95% CI: 85.1–100) for the licensed schedule, 95.8% (95% CI: 92.2–100) for the full dose administered at 0 and 2 weeks, 98.1% (95% CI: 86.9–100) for the full dose administered at 0 and 4 weeks, and 96.1% (95% CI: 83.7–100) for the 3 half doses administered at 0, 2, and 4 weeks (Table 1). All dosing PEP-Vx schedules maintain a high level of predicted survival through week 9 (*36*,*38*).

**FIGURE:**
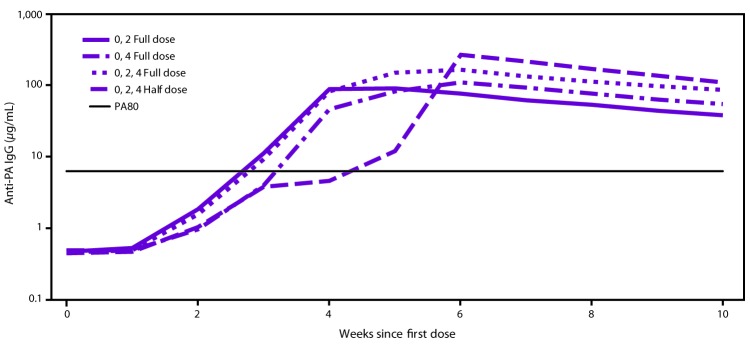
Group geometric means for anti-protective antigen immunoglobulin G enzyme-linked immunosorbent assay over time after administration of anthrax vaccine adsorbed* **Abbreviations:** AVA = anthrax vaccine adsorbed; IgG = immunoglobulin G; PA = protective antigen. * 0, 2 Full dose = 0.5 mL AVA administered at 0 and 2 weeks; 0, 4 Full dose = 0.5 mL AVA administered at 0 and 4 weeks; 0, 2, 4 Full dose = 0.5 mL AVA administered at 0, 2, and 4 weeks; 0, 2, 4 Half dose = 0.25 mL AVA administered at 0, 2, and 4 weeks; PA80 = 80% predicted protection level.

**TABLE 1:** Anthrax vaccine adsorbed postexposure prophylaxis schedules and predicted human survival*

Time from first dose of PEP-Vx	Predicted human survival by PEP-Vx schedule			
	Licensed vaccination regimen	Alternate dose-sparing schedules		
	Full dose at 0, 2, and 4 wks	Full dose^†^ at 0 and 2 wks	Full dose at 0 and 4 wks	Half dose^§^ at 0, 2, and 4 wks
	% (95% CI)	% (95% CI)	% (95% CI)	% (95% CI)
Week 4	95.8 (92.2–100)	95.8 (82.6–100)	72.6^¶^ (58.2–92.9)	91.1 (78.2–98.7)
Week 6	97.4 (85.1–100)	95.5 (81.7–100)	98.1 (86.9–100)	96.1 (83.7–100)
Week 9	96.4 (83.1–100)	93.3 (78.9–100)	97.0 (84.4–100)	94.2 (80.8–100)

**Source:** Data from Stark GV, Sivko GS, VanRaden M, et al. Cross-species prediction of human survival probabilities for accelerated anthrax vaccine adsorbed (AVA) regimens and the potential for vaccine and antibiotic dose sparing. Vaccine 2016;34:6512–7.

**Abbreviations:** AVA = anthrax vaccine adsorbed; CI = confidence interval; PEP-Vx = AVA postexposure prophylaxis.

* Based on survival data from nonhuman primates that received AVA at weeks 0 and 2 and were challenged with a target dose of 200 LD_50_ aerosolized *Bacillus anthracis* spores at week 4.

^†^ 0.5 mL of AVA.

^§^ 0.25 mL of AVA.

^¶^ At 4 weeks before the second dose of vaccine.

#### Duration of Antimicrobial Administration in Combination with Vaccine

Since 2000, FDA has approved several oral antimicrobials (ciprofloxacin in 2000, penicillin G in 2001, doxycycline in 2001, and levofloxacin in 2004) for antimicrobial postexposure prophylaxis (PEP-Abx) of anthrax. Each PEP-Abx regimen should be administered for 60 days when not used in combination with vaccine. However, studies of nonhuman primates have demonstrated that spores can persist in the lungs many days after inhalation exposure (up to 100 days in one animal study) and that anthrax can develop after discontinuation of PEP-Abx (*29*,*30*). Because of the possibility of delayed infection from persistent spores, recommendations for PEP require use of AVA in conjunction with antimicrobial drugs. With this strategy, the antimicrobials protect against germinating spores until the vaccine can elicit a protective immune response.

Using the limited data available at the time, in 2015, FDA licensed a 3-dose regimen of AVA to be given in conjunction with recommended antimicrobials for PEP for persons potentially exposed to aerosolized *B. anthracis* spores (*39*). Newer data from a human clinical trial with AVA demonstrate that, with concurrent receipt of AVA, the duration of antimicrobial use can be shortened from the recommended 60 days (*38*). In the trial, persons were given one of four PEP-Vx regimens: the licensed 3-dose schedule, a dose-sparing schedule with 2 full doses at 0 and 2 weeks, a dose-sparing schedule with 2 full doses at 0 and 4 weeks, or a dose-sparing schedule with 3 half doses at 0, 2, and 4 weeks. Antibody levels predicted to be protective in humans were extrapolated from a matched nonhuman primate nonclinical trial in which 48 nonhuman primates were given 2 doses of AVA at 0 and 2 weeks and challenged with a 200 LD_50_ dose of *B. anthracis* spores at week 4. Protection provided by anti-PA IgG was modeled using logistic regression of the measured prechallenge antibody levels at week 4 in nonhuman primates versus survival of challenge (*36*). The nonhuman primate prediction curve was then applied to the human antibody levels to predict protection in humans (*38*). The estimated peak protection both for licensed and dose-sparing AVA PEP-Vx regimens occurred 2 weeks after the last AVA dose was given. All regimens are estimated to be highly protective; protection is maintained through day 60, when the antimicrobial component of PEP is recommended to end (Table 1).

#### Safety

Since 2008, the vast majority of AVA vaccinations (approximately 8 million doses administered to approximately 1.9 million persons) have been administered by DoD as PrEP to its service members. The PrEP route of administration was SC until FDA approved changing the PrEP route of administration to IM in December 2008. As defined by the U.S. Code of Federal Regulations, an event is classified as serious if one or more of the following conditions is reported: death, a life-threatening illness, hospitalization or prolongation of existing hospitalization, permanent disability, or a congenital anomaly or birth defect (*40*). From January 1, 2009, through June 30, 2017, a total of 2,439 AVA-related adverse events were reported to VAERS (*16*); 329 (13.5%) of these were considered serious. 

During the same period, the 10 most common reported adverse events were coded as headache (14.7%), injection site erythema (13.6%), pain (12.6%), fever (11.6%), fatigue (11.5%), arthralgia (11.2%), erythema (11.2%), injection site pain (9.9%), injection site swelling (9.8%), and rash (9.4%) on the basis of coding terms from the *Medical Dictionary for Regulatory Activities*. VAERS has numerous strengths, such as broad national scope and early detection of possible new, rare, or unusual patterns of adverse events. However, VAERS is a spontaneous reporting system that has important limitations, including underreporting, inconsistent data quality and report completeness, and lack of an unexposed comparison group. Therefore, the data generally cannot be used to assess whether a vaccine caused an adverse event (*16*).

Nine studies, including clinical vaccine trials and observational studies (*10*,*12*,*35*,*37*,*41*–*45*), also assessed serious adverse events after administration of AVA, AV7909, or both in human subjects. Serious adverse events were reported in three studies (*12*,*35*,*43*); however, only in the Anthrax Vaccine Review Program study (*35*) were the serious adverse events considered possibly related to AVA. In this study, 231 adverse events were identified in recipients of approximately 8,300 doses of AVA. Six serious adverse events, none of which was fatal, were considered to be possibly related to the vaccine, including ductal carcinoma of the breast, generalized allergic reaction, new onset bilateral arthralgia of the metacarpal joints associated with positive antinuclear antibody (ANA), bilateral pseudotumor cerebri, supraspinatous tendon tear, and new onset of generalized seizures associated with aqueductal stenosis (*35*).

In studies from the Vaccine Analytic Unit and other groups published since 2010, no association was found between AVA receipt and the following chronic health conditions: reduction in health-related quality of life measures (*46*), multisystem illness (*47*), long-term disabilities (*48*,*49*), type 1 diabetes (*50*), atrial fibrillation (*51*), and diffuse connective tissue diseases (*52*). In one case-control study, an association was identified between AVA receipt and new-onset rheumatoid arthritis if a look-back period of 3 months was used (odds ratio [OR]: 3.93; 95% CI: 1.08–14.27). However, no association between AVA and rheumatoid arthritis was identified if a longer look-back period of 3 years was used (OR: 1.03; 95% CI: 0.48–2.19) (*52*), suggesting that AVA exposure might trigger onset of rheumatoid arthritis in persons who would eventually have developed rheumatoid arthritis later in life.

Although AVA is not intended for use during pregnancy, DoD maintains a registry of women inadvertently vaccinated while pregnant. Studies of adverse outcomes and a cohort study of birth defects using this registry did not detect any increased rates of adverse fetal or infant outcomes among women who received AVA during their first trimester compared with receipt at other time points or no receipt of AVA (*53*,*54*). Finally, because no safety data are available for AVA use in adolescents, a presidential ethics commission proposed comparing AVA safety data for the group aged 18–20 years (the youngest group) and the group aged 21–29 years. If no significant safety difference could be found between the two age groups, then evaluations could proceed in successively younger adolescents. In a study following these suggested methods, AVA was deemed safe in adults aged 18–20 years (*44*); no additional studies have been conducted in younger age groups. AVWG reviewed the VAERS reports and the published literature and presented their findings to the ACIP committee. On the basis of these data, the committee concluded that no clinically significant safety concerns have been identified related to receipt of AVA since 2010 (*9*).

### AV7909

AV7909 is an investigational second-generation anthrax vaccine that is under development for PEP of inhalation anthrax in conjunction with appropriate antimicrobials. AV7909 consists of the licensed AVA combined with a novel adjuvant, CpG 7909, a synthetic immunostimulatory oligodeoxynucleotide. CpG 7909 is a Toll-like receptor 9 agonist that has been demonstrated to augment Th1 responses in humans and enhance innate and adaptive immunity (*7*,*8*)

AV7909 is intended to be added to the Strategic National Stockpile. CDC has submitted a pre–Emergency Use Authorization (EUA) request to FDA to allow potential emergency use of AV7909, in conjunction with appropriate PEP-Abx, for PEP of inhalation anthrax when the supply of the currently licensed AVA is inadequate. EUA is an authority given to the FDA commissioner to legally permit the use of an unapproved medical product or unapproved use of an approved medical product (*55*). Under the proposed EUA, AV7909 would be administered by the IM route as a 2-dose series 2 weeks apart in conjunction with PEP-Abx for adults aged 18–65 years. Pregnant or nursing mothers, older adults (aged ≥66 years), and children (aged <18 years) should receive AVA until data to adequately support AV7909 use in these additional populations under EUA become available. 

Available data indicate that AV7909 might provide the following advantages over AVA:

- Two IM doses of AV7909 administered 2 weeks apart might provide protective immunity 1–2 weeks sooner than the licensed 3-dose PEP-Vx regimen of AVA.
- Compared with the licensed 3-dose AVA PEP-Vx schedule, the 2-dose schedule of AV7909 PEP provides an operational advantage in a large-scale, mass vaccination response.
- Adherence might be better because more persons are likely to complete the 2-dose AV7909 PEP series than the 3-dose AVA PEP series.

#### Immunogenicity

The initial phase 1 clinical trial assessed immunogenicity of AVA alone and AVA plus CpG 7909 in 69 healthy adults aged 18–45 years (*12*). Vaccinations were administered IM on weeks 0, 2 (±1 day), and 4 (±2 days). The CpG group received AVA plus 1 mg of CpG 7909. The results showed that the peak GMT of TNA for the AV7909 group was 8.8-fold higher than that observed for the AVA-alone group; antibody peaked at week 6 in both arms. By week 3, GMT in the CpG group exceeded the peak GMT in the AVA group (reached at week 6). The TNA results paralleled those observed with anti-PA IgG, and in both assays the differences between AVA and AV7909 were statistically significant. Analysis of the phase 2 TNA threshold of protection data for AV7909 revealed that addition of the CpG 7909 adjuvant to AVA improved the kinetics and magnitude of the immune response (*56*). A 2-dose AV7909 regimen with 0.25 mg of CpG 7909 administered IM resulted in a similar serological response at week 9 compared with a 3-dose AVA regimen administered IM and achieved a peak response by week 4 versus week 6 for AVA.

#### Safety

Adverse events were assessed in three clinical trials available at the time of this review, including a total of 241 subjects who were administered at least 1 dose of AVA plus CpG 7909 in varying dose combinations (*10*,*12*,*45*). The most common adverse events, reported in ≥20% of persons receiving AV7909 across these clinical trials, were injection site reactions (e.g., mild to moderate pain, tenderness, and arm motion limitation); these typically resolved within 48 hours of administration. Systemic reactogenicity manifested primarily as mild to moderate fatigue, muscle ache, and headache. No deaths or serious adverse events assessed as being causally associated have been reported in AV7909 clinical studies.

In healthy adults aged 18–50 years who received CpG 7909-adjuvanted experimental vaccines for malaria and hepatitis B in the 0.25-mg dose that is combined with AVA in AV7909 (*57*,*58*), local and systemic reactions were similar to those observed in the groups who received malaria and hepatitis B vaccines without CpG 7909, and the proportion of subjects who dropped out because of adverse events did not differ between treatment and control groups (*58*). Reasons for discontinuation included rash, positive ANA, generalized pruritus, urticaria, and fever. These clinical trial reports suggested that these adverse events might be a result of activation of proinflammatory innate immune responses at the injection site. Theoretically, CpG 7909 could trigger the onset of autoimmune disease, possibly as a result of nonspecific T or B lymphocyte activation. Some studies reported mild to moderate increases in anti–double-stranded DNA antibody, rheumatoid factor, or positive ANA results. However, these increases in immune markers were typically transient. No adverse events suggesting autoimmune disease have been reported in the reviewed published data on CpG 7909-adjuvanted infectious disease vaccine trials (*57*–*61*). No safety data are available for CpG 7909-adjuvanted vaccines, including AV7909 among special populations (e.g., children, persons aged >65 years, and pregnant women).

## Recommendations for Prevention of Anthrax Among Persons with Potential Risk for Exposure: PrEP

ACIP previously recommended AVA PrEP for prevention of anthrax in persons at high risk for exposure to *B. anthracis* (e.g., members of the U.S. military deployed to areas designated by DoD as high risk for exposure, laboratory workers who work with high concentrations of *B. anthracis,* and persons such as farmers, veterinarians, and livestock handlers who might handle infected animals or contaminated animal products) (*9*). In this report, ACIP recommends that a booster dose of AVA PrEP be given every 3 years to persons who are not at high risk for exposure to *B. anthracis* who have previously completed the 3-dose primary and the initial 2-dose boosters AVA series and want to maintain protection.

The PrEP schedule for persons at high risk for exposure to *B. anthracis* is AVA administered IM as a priming series at 0, 1, and 6 months, with booster doses at 12 and 18 months and annually thereafter. If the vaccination schedule is interrupted, the series does not need to be restarted. After the priming series is completed, persons can work in high-risk areas of exposure with appropriate personal protective equipment and biosafety measures. Documentation of seroconversion is not required. If biosafety or respiratory protection measures are breached and exposure to aerosolized *B. anthracis* spores might have occurred, a 30-day course of PEP-Abx is recommended, regardless of whether PrEP has been fully or partially completed.

Because of the lack of a quantifiable risk, emergency and other responders are not recommended to receive routine PrEP vaccination. However, emergency responders, because of the requirements of their occupation, might be exposed to aerosolized *B. anthracis* spores and thus may opt to receive the vaccine on a voluntary basis. For persons who are not currently at high risk for exposure to *B. anthracis* but who might be at high risk in the future (e.g., persons involved in emergency response activities), published data (*35*) support a booster dose interval of >1 year. Thus, a booster dose of AVA can be given every 3 years to persons not at high risk for exposure to *B. anthracis* who have previously received the initial AVA priming and booster series and who want to maintain protection. After completing the initial 3-dose priming and booster series, persons who have not received a booster dose in the last 12 months and need to enter an area where *B. anthracis* is suspected to be present in the environment or be in use should be given an IM booster dose and then either wait 2 weeks to enter the high-risk area or, if required to enter immediately, take PEP-Abx for 2 weeks. While in a high-risk area, a booster dose should be given within 1 year of the last booster dose.

Persons who are exposed to aerosolized *B. anthracis* spores but have not completed the initial priming and booster series for AVA should receive additional AVA doses and PEP-Abx. The number of vaccine doses and duration of PEP-Abx will vary in a manner commensurate with the number of previously received doses (Table 2).

**TABLE 2:** Transition from preexposure prophylaxis* schedule to postexposure prophylaxis schedule for persons who have not completed a priming and initial booster series^†^ and must immediately enter an area that poses a high risk^§^ for *Bacillus anthracis* exposure

Previous PrEP doses	Interval since last dose	PEP	
		PEP-Vx	PEP-Abx^¶^
0	—	Dose 1 (week 0)	Administer until 42 days after first dose of AVA or 14 days after last dose, whichever occurs later.
		Dose 2 (week 2)	
		Dose 3 (week 4)	
1	—	Dose 2 (week 0)	Administer until 28 days after second dose of AVA or 14 days after the last dose, whichever occurs later.
		Dose 3 (week 2)	
2	—	Dose 3 (week 0)	Administer until 14 days after last dose.
3, 4	>6 mos	Booster dose	Administer until 14 days after booster dose.
3, 4	≤6 mos	No booster	No antimicrobials needed

**Abbreviations:** AVA = anthrax vaccine adsorbed; PEP = postexposure prophylaxis; PEP-Abx = antimicrobial PEP; PEP-Vx = AVA PEP; PrEP = preexposure prophylaxis.

*No data are available on the effect on the immune response for starting PrEP by the intramuscular route and switching to the subcutaneous route to join the PEP schedule; however, no evidence suggests that the immune response would be adversely affected by mixing the routes of administration.

^†^ Priming doses at 0, 1, and 6 mos, with booster doses at 12 and 18 mos.

^§^ The licensed booster schedule for high-risk exposure applies while in the high-risk area.

^¶^ If the AVA series cannot be completed, then antimicrobial therapy should continue for 60 days.

## Recommendations for Prevention of Anthrax Among Persons with Suspected or Known Exposure: PEP 

ACIP recommends AVA for use in adults aged 18–65 years to be given in conjunction with a course of antimicrobials (Table 3) to prevent infection after suspected or known exposure to aerosolized *B. anthracis* spores. Antimicrobial duration details are provided (Table 4). The vaccine is given at a dose of 0.5 mL SC at 0, 2, and 4 weeks postexposure, unless the emergency response requires a change to the IM route or use of dose-sparing regimens. If the PEP-Vx schedule is interrupted, the series does not need to be restarted. Instead, subsequent doses should be administered as soon as possible, and the series should be finished.

**TABLE 3:** Oral antimicrobial dosages for use in adults in conjunction with anthrax vaccine adsorbed for postexposure prophylaxis

Strain	Drug and dosage*
For all strains, regardless of penicillin susceptibility or if susceptibility is unknown	Ciprofloxacin,^†^ 500 mg every 12 hrs
	Doxycycline,^†^ 100 mg every 12 hrs
	Levofloxacin, 750 mg every 24 hrs
	Moxifloxacin,^§^ 400 mg every 24 hrs
	Clindamycin,^§^ 600 mg every 8 hrs
Alternatives for penicillin-susceptible strains	Amoxicillin,^§^ 1,000 mg every 8 hrs
	Penicillin VK,^§^ 500 mg every 6 hrs

**Abbreviations:** FDA = Food and Drug Administration; PEP-Abx = antimicrobial postexposure prophylaxis.

* Any one of these drug regimens.

^†^ First-line drugs; alternative drugs are listed in order of preference for PEP-Abx for patients who cannot take first-line treatment or if first-line PEP-Abx is unavailable.

^§^ Not FDA approved for PEP-Abx of inhalation anthrax.

**TABLE 4:** Antimicrobial duration when used in conjunction with Food and Drug Administration–licensed or dose-sparing postexposure prophylaxis regimens of anthrax vaccine adsorbed*

Population with suspected or known exposure	Duration of antimicrobial regimen
Immunocompetent adults aged 18–65 yrs	42 days when initiated concurrently with first dose of AVA or for 14 days after last AVA dose, whichever is later (not to exceed 60 days)
Adults aged 18–65 yrs with immunocompromising conditions (e.g., cancer or HIV infection) or receiving immunosuppressive therapy (e.g., high-dose corticosteroids for >2 wks or radiation therapy)^†^	60 days
All older adults (>65 yrs)	60 days
All pregnant women and nursing mothers	60 days
All children (≤17 yrs)	60 days

**Abbreviation:** AVA = anthrax vaccine adsorbed.

* If the AVA series cannot be completed, then antimicrobial therapy should continue for 60 days.

^†^ **Source: **Löbermann M, Boršo D, Hilgendorf I, Fritzsche C, Zettl UK, Reisinger EC. Immunization in the adult immunocompromised host. Autoimmun Rev 2012;11:212–8.

### Route of Administration

ACIP recommends the SC route of administration rather than the IM route for PEP because higher antibody concentrations are achieved by 4 weeks after AVA vaccination. However, during a large-scale emergency response, AVA for PEP can be administered using an IM route if the SC route of administration poses significant materiel, personnel, or clinical challenges that might delay or preclude vaccination. In addition, persons who experienced adverse events from AVA that was administered SC may elect to receive subsequent vaccine doses IM after consultation with a health care provider. Doses of AVA inadvertently administered by the IM route rather than the SC route do not need to be repeated by the SC route.

### Dose-Sparing PEP Regimens

ACIP recommends use of dose-sparing PEP regimens if the anthrax vaccine supply is insufficient to vaccinate all potentially exposed persons. The 2 full-dose strategy will expand the existing vaccine supply by 50%, and the 3 half-dose strategy will expand the supply by 100%. Immediately after a wide-area aerosolized release of *B. anthracis* spores, the preferred dose-sparing PEP regimen might not be apparent until the size of the event is determined. All dose-sparing PEP-Vx regimens are estimated to provide high levels of protection 2 weeks after the last dose (Table 5). Existing data indicated that 2 doses administered 2 weeks apart or 4 weeks apart are effective; therefore, the 2-dose schedule should be ≥2 weeks apart and ≤4 weeks apart, recognizing that full protection is not achieved until 2 weeks after the second dose (*37*).

**TABLE 5:** Postexposure prophylaxis with anthrax vaccine adsorbed dose-sparing regimens

Dose	Route of administration	Dosing schedule
0.5 mL (full dose)	SC or IM*	2 doses: 0 and 2–4 wks
0.25 mL (half dose)	SC or IM*	3 doses: 0, 2, and 4 wks

**Abbreviations:** IM = intramuscular; SC = subcutaneous.

*Can be administered IM if the SC route of administration poses significant materiel, personnel, or clinical challenges that might delay or preclude vaccination.

### Antimicrobial Duration in Conjunction with FDA-Licensed or Dose-Sparing PEP Regimens of AVA

ACIP recommends that in immunocompetent adults (e.g., healthy, nonpregnant adults aged 18–65 years), PEP-Abx both for the licensed and dose-sparing PEP-Vx regimens can be discontinued 42 days after initiation of vaccine if AVA is administered on schedule for both the licensed and dose-sparing PEP-Vx regimens (Table 4). If the AVA series cannot be completed, then antimicrobial therapy should continue for 60 days. However, the second dose of AVA is critical for producing high antibody concentrations. To account for delays in initial vaccination that might occur because of the emergency situation, antimicrobial administration should be initiated as soon as possible and continued for 42 days after the first dose or 2 weeks after the last dose of the vaccine series, whichever comes last. No data on humans are available to suggest that PEP-Abx should be given for >60 days, which is the recommended duration for PEP-Abx when given without vaccine. Thus, PEP-Abx should not be given for >60 days, regardless of the timing of last vaccine dose. 

The shortening of PEP-Abx duration from 60 days to 42 days, or 2 weeks after the last dose of vaccine, applies to healthy adults aged 18–65 years. Persons with immunocompromising conditions that might interfere with their ability to develop an adequate immune response or populations for whom data on immune response to AVA are lacking (e.g., children, pregnant women, and adults aged ≥65 years) should continue to receive PEP-Abx for 60 days concurrently with AVA.

### Potential Emergency Use of AV7909

Because of supply concerns and the investigational status of AV7909, AVA should be prioritized over AV7909 for PEP-Vx of potential exposure to aerosolized *B. anthracis* spores. However, the limited amount of phase 2 safety and immunogenicity data indicate that AV7909 appears to be safe and effective. The benefits of an effective vaccine that can prevent anthrax outweigh the known potential risks for adverse events in persons potentially exposed to aerosolized *B. anthracis* spores. Therefore, if supplies of AVA are exhausted or unavailable, AV7909 is an option for PEP of persons exposed to aerosolized *B. anthracis* spores under an EUA granted by FDA. As with AVA, antimicrobials (Tables 3 and 4) should be taken in conjunction with AV7909. Additional AV7909 data on safety, immunogenicity, and biocompatibility with antimicrobials will be reviewed by ACIP as they become available, and recommendations on potential preferential use will be updated as needed.

No data are available on the immunogenicity or safety of AV7909 for children or other special populations. However, a phase 2 clinical trial is being conducted to assess the safety and immunogenicity of AVA and AV7909 in adults aged >65 years compared with adults aged 18–50 years (*62*). In the absence of such data, AVWG considered it reasonable to anticipate that risks and benefits of PEP-Vx for children or special populations would be similar to those for the general adult population. Therefore, if AVA is not available, emergency use of AV7909 under an appropriate regulatory mechanism should be considered for all populations with known or potential exposure to aerosolized *B. anthracis* spores. Should an anthrax exposure event occur that necessitates AV7909 use while it remains under development and is not yet licensed, ACIP will convene an emergency meeting to review available data for specific recommendations on AV7909 emergency use.

No studies have been conducted on the interchangeability of AVA and AV7909. When feasible, doses of the same vaccine type should be used to complete a series. However, vaccination should not be deferred because the previously used vaccine type is unavailable. When a vaccine series uses a combination of AVA and AV7909, 3 total doses of anthrax vaccine should be administered and used in conjunction with appropriate antimicrobials (Tables 3 and 5).

### Antitoxin Use for PEP

Three licensed anthrax antitoxins are available from the Strategic National Stockpile: anthrax immune globulin intravenous (AIGIV) (*63*), obiltoxaximab (Anthim) (*64*), and raxibacumab (ABthrax) (*65*). AIGIV is a polyclonal antibody, whereas obiltoxaximab and raxibacumab are both monoclonal antibodies. All work by binding to protective antigen, which blocks movement of toxins into cells and therefore the effects of toxins within the cells. All three antitoxins are indicated in all adults and children for the treatment of inhalation anthrax due to *B. anthracis,* in combination with appropriate antimicrobial drugs. 

Obiltoxaximab and raxibacumab also have an indication for PEP of inhalation anthrax due to *B. anthracis* when alternative therapies are not available or are not appropriate. In these situations, obiltoxaximab or raxibacumab may be considered to help prevent inhalation anthrax. The predicted effectiveness of both antitoxins for this indication is based solely on efficacy studies conducted in animal models of inhalation anthrax (*66*,*67*).

Data indicate that raxibacumab can be coadministered with AVA for PEP without affecting vaccine immunogenicity (*68*). No data are available to assess whether obiltoxaximab coadministered with AVA impairs vaccine immunogenicity. AIGIV does not have a PEP indication because coadministration of AIGIV and AVA in a rabbit model has been shown to significantly reduce the development of an immune response to AVA (*68*).

## Vaccine Adverse Events Reporting and Additional Information

Surveillance for serious adverse events is important for all antimicrobials, biologics, and vaccines. All clinically significant adverse events after receipt of antimicrobials or anthrax antitoxin for PEP or treatment of anthrax should be reported to the MedWatch Program (https://www.fda.gov/safety/medwatch-fda-safety-information-and-adverse-event-reporting-program or 888-463-6332). All clinically significant adverse events after receipt of either AVA or AV7909 should be reported to VAERS (https://vaers.hhs.gov or 800–822–7967). Additional information about anthrax and anthrax vaccines is available at https://www.cdc.gov/anthrax.

## Future Directions

Research priorities for future studies on anthrax vaccines should include assessment of immunogenicity and safety in special populations, such as children, older adults, and pregnant and nursing mothers; additional evaluations of the dose-sparing schedules; evaluation of the interchangeability of AVA and AV7909; determination of the optimal booster schedule to provide long-term protection after receiving the PEP vaccine schedule; testing of the stability of AVA and AV7909 outside the cold chain; assessment of whether coadministration of obiltoxaximab with AVA impairs vaccine immunogenicity; and the optimal duration of antimicrobial use in postexposure settings. Studies are planned to evaluate the effect of longer intervals between PrEP boosters on vaccine responses. On approval of AV7909 as a licensed vaccine, the additional data leading to the licensure of AV7909 will be reviewed by ACIP, and recommendations will be updated as needed.
